# Federated k-means based on clusters backbone

**DOI:** 10.1371/journal.pone.0326145

**Published:** 2025-06-12

**Authors:** Zilong Deng, Yizhang Wang, Mustafa Muwafak Alobaedy

**Affiliations:** 1 College of Information Technology, Anqing Vocational and Technical College, Anqing, China; 2 Faculty of Information Technology, City University Malaysia, Petaling Jaya, Malaysia; 3 College of Information Engineering, Yangzhou University, Yangzhou, China; Najran University College of Computer Science and Information Systems, SAUDI ARABIA

## Abstract

Federated clustering is a distributed clustering algorithm that does not require the transmission of raw data and is widely used. However, it struggles to handle Non-IID data effectively because it is difficult to obtain accurate global consistency measures under Non-Independent and Identically Distributed (Non-IID) conditions. To address this issue, we propose a federated k-means clustering algorithm based on a cluster backbone called FKmeansCB. First, we add Laplace noise to all the local data, and run k-means clustering on the client side to obtain cluster centers, which faithfully represent the cluster backbone (*i.e.*, the data structures of the clusters). The cluster backbone represents the client’s features and can approximatively capture the features of different labeled data points in Non-IID situations. We then upload these cluster centers to the server. Subsequently, the server aggregates all cluster centers and runs the k-means clustering algorithm to obtain global cluster centers, which are then sent back to the client. Finally, the client assigns all data points to the nearest global cluster center to produce the final clustering results. We have validated the performance of our proposed algorithm using six datasets, including the large-scale MNIST dataset. Compared with the leading non-federated and federated clustering algorithms, FKmeansCB offers significant advantages in both clustering accuracy and running time.

## 1 Introduction

The k-means clustering algorithm is a widely employed unsupervised learning method aimed at partitioning *n* samples in a dataset into *k* clusters such that the samples within each cluster are as similar as possible. In contrast, samples from different clusters are as dissimilar as possible. This similarity is typically measured by the distance between sample points. The steps of k-means are as follows: (1) random initialization: randomly select *k* points from the dataset to serve as the initial cluster centers. (2) assignment: for each data point in the dataset, assign it to the cluster whose center is closest to it based on the distance measure. (3) update cluster centers: recalculate the mean (or centroid) of all points in each cluster and use this mean as the new cluster center. (4) iteration: repeat steps (2) and (3) until the cluster centers no longer significantly change or a predetermined number of iterations is reached, indicating convergence. k-means has two advantages: (1) simplicity and intuitiveness: The algorithm is straightforward to understand and implement [1]. (2) efficiency: it performs well on large datasets, particularly when the clusters have roughly equal density [2].

The k-means clustering algorithm has a wide range of applications in multiple fields [3]. For example, in market segmentation, the k-means algorithm can divide customers into different groups based on consumer purchasing behavior [4], preferences, and other data, helping enterprises implement refined marketing strategies; in the field of image processing [5], the k-means algorithm can be used for image segmentation, clustering image pixels according to color or texture features to achieve automatic image segmentation; in bioinformatics [6], the k-means clustering algorithm can be used to discover gene clusters with similar expression patterns, which is conducive to understanding gene function and regulatory networks.

The k-means clustering algorithm has several drawbacks, which can be summarized as follows: (1) sensitivity to initial cluster centers: The k-means algorithm is sensitive to the choice of initial cluster centers. Different initializations can lead to different clustering results, which may or may not represent the true underlying structure of the data. This can result in the algorithm converging to a local optimum rather than a global optimum [7]. (2) need for a predetermined number of clusters (*k*): The algorithm requires the user to specify the number of clusters (*k*) in advance. Determining the optimal number of clusters can be challenging, especially for complex or high-dimensional datasets [8]. (3) assumption of spherical clusters: k-means assumes that clusters are spherical and of similar size and density. This may not always hold true in real-world datasets, where clusters can have complex shapes and varying densities [9, 10]. (4) sensitivity to outliers: outliers, or data points that are significantly different from the rest of the data, can dramatically impact the clustering results. k-means is not robust to outliers and can assign them to clusters based solely on their proximity to the cluster centers [11]. (5) scalability issues: While k-means can be parallelized to some extent, it can still be computationally expensive for very large datasets. The time complexity of the algorithm increases with the number of data points and iterations required for convergence [12]. (6) non-deterministic results: Due to its sensitivity to initial cluster centers, the k-means algorithm can produce different clustering results each time it is run on the same dataset. This can make it difficult to reproduce results and compare them across different runs [13]. (7) limited interpretability: While k-means can effectively partition data into clusters, it does not provide insights into the factors that define those clusters or their relationships. Further analysis may be required to understand the characteristics of the clusters and their significance.

There are many works improving k-means from the previous seven issues: For example, Nie *et al*. [14] proposed k*-means clustering algorithm, by incorporating the hierarchical optimization principle, cluster pruning strategy, and optimized update theory, effectively addresses the issues of traditional k-means algorithms, including sensitivity to initial centroid selection, vulnerability to outliers and noise points, and slow convergence on large-scale datasets. Experimental results demonstrate that this method exhibits superior performance across various datasets, offering new insights and approaches for the research and application of k-means clustering algorithms. The traditional k-means algorithm faces two major issues in unsupervised learning: firstly, it requires the specification of the optimal number of clusters (i.e., the *k* value), which is often challenging to accurately estimate in practical applications; secondly, the clustering outcomes are significantly influenced by the initial selection of cluster centers, potentially leading to local optima rather than global optima. To address these limitations, Sinaga *et al*. proposed improvements to the k-means algorithm, aiming to develop an unsupervised k-means clustering algorithm that can automatically determine the optimal number of clusters and produce more stable clustering results [15]. To overcome the limitations of the traditional k-means algorithm, Hu *et al*. proposed a novel k-means clustering algorithm with major improvements including: Automatic determination of the optimal number of clusters *k*: By utilizing certain optimization strategies (such as the elbow method, silhouette coefficient method, etc.) or incorporating the inherent characteristics of the data, the algorithm automatically identifies the optimal number of clusters *k*, thereby reducing human intervention. Optimized selection of initial cluster centers: A more scientific approach (such as the k-means++ algorithm) is employed to select the initial cluster centers, ensuring that they are representative and consequently enhancing the stability and accuracy of the clustering results. Enhanced adaptability and scalability of the algorithm: Tailored to the characteristics of big data, the algorithm optimizes its data processing flow and storage methods, improving its efficiency and stability when handling large-scale datasets [16]. Robust deep k-means aims to address the limitations that traditional k-means clustering algorithms encounter when dealing with complex and high-dimensional data, such as being prone to local optima, sensitivity to initial cluster center selection, and the difficulty in automatically determining the optimal number of clusters (*k*). Additionally, conventional clustering methods typically rely on the raw data representation, which can become ineffective when data points reside in high-dimensional spaces. This phenomenon is often referred to as the “curse of dimensionality” [17]. The k-means are often difficult to interpret, partly because they intricately rely on all features of the data. To improve upon this, Moshkovitz *et al*. proposed using small decision trees to partition the dataset into clusters, thereby representing the clusters in a more straightforward manner [18].

However, there are relatively few efforts that simultaneously focus on the scalability and privacy protection of k-means. Especially in the era of big data, the scalability of algorithms and the protection of user information are two crucial issues. In recent years, the development of federated learning has provided new inspiration to the clustering algorithm community, leading to the emergence of federated clustering.

Federated clustering is a distributed clustering algorithm under privacy constraints. The following is a detailed explanation of traditional federated clustering: The definition of federated clustering is as follows: Federated clustering utilizes the framework of federated learning, allowing clients to process their respective data locally and share necessary information through secure communication protocols to collaboratively complete clustering tasks. Background: In traditional clustering algorithms, all data typically needs to be centrally stored for processing, which may lead to risks of data privacy leakage. Federated clustering effectively mitigates this issue through distributed computing and data isolation. In federated clustering, data is distributed across different clients, with each node holding only a portion of the data. Privacy Protection: During the clustering process, each node only processes data locally. Through iterative information exchange and clustering calculations, nodes work together to ultimately obtain globally consistent clustering results. While specific federated clustering algorithms may vary, the general process includes the following steps: (1) Initialization: Each local client performs initial clustering operations on its own data to determine initial cluster centers. (2) Local Clustering: Each client performs local clustering on its data based on the initial cluster centers, calculates the distance between data points and cluster centers, and assigns data points to the nearest cluster center. (3) Information Exchange: clients exchange necessary clustering information, such as updates to cluster centers or data point distributions, through secure communication protocols. (4) Cluster Center Updates: Based on the exchanged information, each node updates its own cluster centers. Iterative Convergence: Repeat the above steps until the clustering results converge or a preset number of iterations is reached. (5) Global Clustering Results: When the algorithm converges, each node aggregates its local clustering results to obtain globally consistent clustering results.

In this paper, we propose a new Federated K-means based on Cluster Backbone called FKmeansCB. The idea is simple, in local clients, we add Laplace noise to all the local data and use cluster centers generated by k-means to represent the data points within the cluster, cluster centers are the centroids of the entire cluster. All the centers form the cluster backbone of local data in the clients, which represents the whole structure information of local data. We upload the cluster centers to the server, *i.e.,* upload structure information of local clients to the server, the server obtains structure information of all the local clients only using uploaded cluster centers. Naturally, the server can obtain global similarity measures in the Non-Independent and Identically Distributed (Non-IID) data environments and better federated clustering results will be obtained. The contributions of this paper are as follows:

(1) We propose a new federated k-means algorithm with time complexity *O*(*n*).

(2) The proposed federated k-means performs better than the state-of-the-art federated and non-federated clustering algorithms.

The rest of this paper is organized as follows. We describe the traditional federated clustering framework and the new proposed federated k-means in Sect 2. In Sect 3, we perform extensive experiments to compare FKmeansCB with four benchmark models and analyze the experimental results in Sect 4. Finally, in Sect 5, we conclude our paper and explore future work.

## 2 Materials and methods

### 2.1 The traditional federated clustering framework

Federated clustering is a technique that combines federated learning and clustering algorithms, aimed at performing clustering analysis on distributed data while protecting data privacy [19, 20]. The emergence of this method provides new solutions for processing large-scale, sensitive, or distributed datasets.

Federated clustering has broad application prospects in fields such as healthcare, finance, and the Internet of Things, especially when dealing with sensitive data involving personal privacy. Despite its many advantages, federated clustering also faces challenges such as communication costs, computational efficiency, and data heterogeneity. Additionally, ensuring data privacy and security during the clustering process is also an important issue.

**Fig 1:**
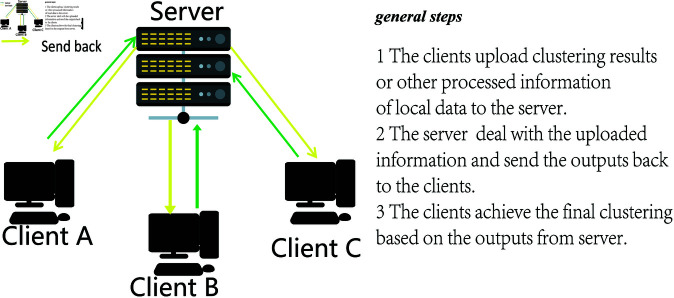
A basic federated clustering framework.

A basic federated clustering framework is shown in Fig 1, the clients and a server form a federated clustering system. Each client trains a local clustering algorithm and uploads the clustering results or other processed output to the server, the server aggregates all the uploaded data generates new information, and sends them back to clients, and each client achieves final clustering according to the received information. In federated clustering, local data do not leave the client, so data privacy is enhanced.

The core of federalization lies in a distributed learning approach, thereby making federated clustering fundamentally a distributed clustering algorithm that prioritizes data security. This algorithm inherently possesses the capability to manage big data. In contrast to traditional distributed clustering, data distribution in a federated environment is often NoN-IID due to its potential sourcing from a range of portable devices and terminals. The confluence of NoN-IID and the prohibition on direct transmission of raw data account for the current suboptimal performance of federated clustering algorithms. Thus, how to improve the clustering accuracy for NoN-IID is an important issue in the field of federated clustering.

We introduce three important improved k-means clustering algorithms as follows: (1) Nie’s work: the conventional k-means algorithm aims to minimize the sum of squared distances, Nie’s work reframes this objective as a trace maximization problem, which involves maximizing the trace (sum of diagonal elements) of a matrix, a common concept in optimization and matrix theory. By the way, Nie’s work is a centralized clustering algorithm [14]. (2) k-FED: each client independently runs a variant of Lloyd’s algorithm, and all clients send their local clustering centers to the server. The server selects one of the device’s cluster centers as the initial centers and then selects the farthest client cluster centers to be merged into the initial centers until *k* cluster centers are selected. *k* centers serve as global centers to guide all client data clustering [21]. (3) MUFC: SOTA federated clustering algorithm published in ICLR2023 [22]. Each client runs k-means++ to group local data, and at the server, MUFC uses a proposed secure compressed multiset aggregation method to combine all the local clustering results.

The proposed FKmeansCB differs significantly from the three algorithms mentioned above. k-FED and MUFC are very different from FKmeanCB in the final cluster center selection at the server. k-FED selects all local cluster centers of a client as part of the global cluster center, MUFC uses a secure compressed multiset aggregation strategy to obtain the class center, while FKmeanCB performs k-means on all local class centers to obtain the global class center. In terms of local data protection strategy, k-FED adopts approximate local cluster center, MUFC adopts uniform quantification, and FKmeansCB adopts localized differential privacy. K-FED may not be able to effectively capture the intrinsic structure and similarity of data. The assumption that the number of clusters on each client is less than the square root of the total number of clusters may not hold in complex Non-IID scenarios, leading to poor clustering performance. In Non-IID scenarios, the data structures of different clients vary greatly. MUFC may not be able to effectively integrate these different feature information, making it difficult to capture key features that represent global data, and thus unable to accurately partition data points into appropriate clusters. FKmeansCB uses cluster backbone to obtain structures of different clients and achieves better performance on Non-IID data.

### 2.2 FKmeansCB

For the k-means clustering algorithm, the data points allocation mechanism is to assign them to the nearest centrality to form clustering results, the allocation mechanism is not affected by Non-IID distributions because it does not need to consider the nearest neighbor relationships between data points across different clients. However, at the server, the global center’s decision of k-means requires dependency on data across different clients, according to the definition of federated clustering, local data cannot leave the client, the clients only can upload processed information to the server. The server can not use all the local data (incomplete global similarity) to calculate the global centers, which may reduce the clustering accuracy. The method to improve the accuracy of federated clustering is very simple: just find a method that can approximate the global similarity at the server, to obtain more accurate cluster centers and obtain high-quality federated clustering results. Based on the above idea, in this paper, we propose a new federated k-means clustering algorithm, namely the federated k-means clustering algorithm based on cluster backbone.

Clusters refer to clusters formed after data clustering, that is, a set of similar data points [23]. The backbone can be understood as a core structure or main component. For example, in hierarchical clustering algorithms, the clustering results may form a tree structure, where the main branches or key connecting parts can be seen as the cluster backbone, which represents the core architecture of the clustering results and helps to understand the overall distribution of data and the relationships between clusters.

Therefore, the key to solving the problem is how the client can maximize the use of its data, obtain appropriate information, and send it to the server. Considering the characteristics of the k-means clustering algorithm, the final cluster center is the centroid of all data points within the cluster. The cluster center can best represent the geometric characteristics of a cluster. For a client, all cluster centers obtained by k-means can express the cluster backbone (i.e. data structure information) of the client’s data points. Therefore, we propose that each client use the clustering algorithm to find the cluster center and pass it to the server. In this way, in the next stage, the server can obtain global data structure information, making it easier to find an approximate global cluster center. The process of the proposed new federated k-means clustering algorithm based on clusters backbone is as follows:

First, we train a local k-means clustering algorithm. We first add Laplace noise ($Lap(\frac{1}{\epsilon },0)$) to all the local data, where $Lap(\frac{1}{\epsilon },0)$ denotes random Laplacian noises generated by Laplacian distribution: $Lap(\lambda ,\mu )=\frac{1}{2\lambda }e^{-(x-\mu )/\lambda }$. Regardless of the volume of data locally, the time complexity of k-means clustering remains *O*(*n*), which is one of the lowest in known clustering algorithms. In k-means, the parameter *k1* denotes both the number of initial cluster centers and the resulting number of clusters. The *k1* final cluster centers are the centroids of all data points within their clusters. The cluster centers generated by k-means inherently reflect the structure of the cluster clusters. Consequently, these cluster centers encode local data structure information. Thus, we can represent large datasets with just a fraction of the data, significantly reducing time complexity and enhancing the scalability of FKmeansCB. Subsequently, each client uploads the *k1* cluster centers to the server, allowing the server to aggregate comprehensive data structure information from all clients.

Secondly, the server aggregates all sent client cluster centers, thereby obtaining comprehensive client data structure information. This enables the server to derive the global data structure by utilizing a subset of the dataset, rather than requiring all original data points. Subsequently, the server continues to run k-means to obtain the *k2* global cluster centers. These cluster centers, derived from the global data structure information, are more accurate than those calculated in NoN-IID environments. The parameter *k1* denotes the number of clusters in the clients, and *k2* denotes the number of clusters in k-means at the server. Once the cluster centers are determined, the server sends them to all clients.

Finally, after receiving the global cluster center, the client uses the simplest method to obtain the final clustering result, which is to allocate all local data points to the nearest cluster center.

**Fig 2:**
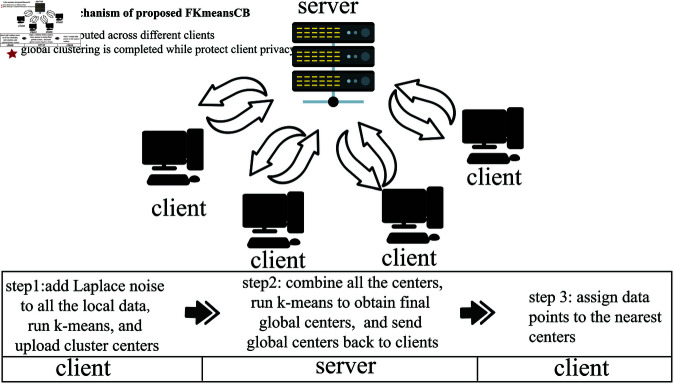
The framework of the proposed FKmeansCB.

To provide a more intuitive explanation of the algorithm process, we present an algorithm flowchart as shown in Fig 2 and an algorithm flowchart as shown in Algorithm 1.

**Algorithm 1. The proposed algorithm.**

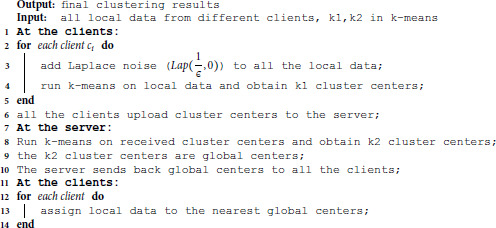



For Algorithm 1, lines 1–5 are client-side algorithms, where each client receives *k* cluster centers through the execution of k-means. Lines 7–10 represent the server-side algorithm. Using the k-means algorithm again to cluster all the cluster centers uploaded by the client, *k1* global centers were obtained. Lines 12–14 represent the client-side algorithm, which completes the final clustering task.

### 2.3 Time complexity analysis

See Algorithm 1, in lines 1–4, the time complexity of k-means is *O*(*n*), where *n* is the number of all data points. On the server side, the algorithm’s time complexity is *O*(*m*), where *m* is the number of centers in all clients and *m*<<*n*. For rows 11–13, the final clustering time complexity is *O*(*p*), where *p* is the number of client data points and *p*<<*n*. Therefore, the total time complexity of the proposed algorithm is O(n+m+p)≈O(n), where *m*<<*p*<<*n*. The time complexity of the proposed algorithm is lower than most known federated clustering algorithms.

## 3 Results

In this paper, see Table 1, we use six datasets to evaluate the proposed algorithms: Thyroid, Breast, Ecoli, Iris, Covertype, and MNIST. The Covertype and MNIST datasets are large-scale datasets, Covertype has 581,012 samples, and MNIST has 70,000, so it is hard to clustering them for most clustering algorithms. The used datasets are non-federated, we use the method of [22] to change them into federated datasets. Especially for Covertype and MNIST, we use the UMAP to reduce the dimensionality by 2. All the algorithms run on the same machine. In addition, we use popular ARI and NMI to evaluate all the clustering results. We run the algorithms 20 times with different parameter values (see Table 2, parameter values are in parentheses) and report the best one in this paper.

**Table 1:** Dataset features.

ID	Datasets	#Samples	#Dimensions	#Natural clusters
1	Thyroid	215	5	3
2	Breast	277	9	2
3	Ecoli	336	7	8
4	Iris	150	4	3
5	Covertype	581,012	54	7
6	MNIST	70,000	784	10

**Table 2:** Comparison between the proposed algorithm and other clustering methods on six datasets (parameter values are in the parentheses).

	Thyroid		Ecoli	
	ARI	NMI	ARI	NMI
k-means	0.58(3)	0.49(3)	0.39(3)	0.58(3)
Nie’s work	0.33(5)	0.26(5)	0.63(3)	0.67(3)
k-FED	0.43(3/1)	0.54(3/1)	0.61(4/2)	0.62(4/2)
MUFC	0.58(3/3)	0.47(3/3)	0.67(3/3)	0.64(3/3)
**FKmeansCB**	**0.60(7/3)**	**0.51(7/3)**	**0.71(11/3)**	**0.68(11/3)**
	**Breast**		**Iris**	
	**ARI**	**NMI**	**ARI**	**NMI**
k-means	0.17(2)	0.09(2)	0.73(3)	0.76(3)
Nie’s work	0.06(3)	0.07(3)	0.72(2)	0.74(2)
k-FED	0.16(2/1)	0.08(2/1)	0.73(4/1)	0.75(4/1)
MUFC	0.17(2/2)	0.09(2/2)	0.73(3/2)	0.77(3/2)
**FKmeansCB**	**0.20**(9/2)	**0.10**(9/2)	**0.75**(5/3)	**0.80**(5/3)
	**MNIST**		**Covertype**	
	**ARI**	**NMI**	**ARI**	**NMI**
k-means	0.39(10)	0.50(10)	0.01(7)	0.07(7)
Nie’s work	NaN	NaN	NaN	NaN
k-FED	0.75(10/2)	0.84(10/2)	0.04(7/2)	0.13(7/2)
MUFC	0.77(10/8)	0.85(10/8)	0.03(5/4)	0.12(5/4)
**FKmeansCB**	**0.88**(200/10)	**0.89**(200/10)	**0.05**(200/10)	**0.15**(200/10)

The NMI and ARI index is defined as follows:

$$NMI(X,Y)=\frac{2MI(X,Y)}{H(X)+H(Y)},$$ (1)

where *X* represents the non-duplicate value of the real label, and *Y* represents the non-duplicate value of the cluster label, *H*(*X*) is the entropy of *X*, *H*(*Y*) is the entropy of *Y*, *MI*(*X*,*Y*) defined as:

$$MI(X,Y)=\sum_{i=1}^{\left|X\right|}\sum_{j=1}^{\left|Y\right|}P(i,j)log\frac{P(i,j)}{P(i)P(j)},$$ (2)

where *P*(*i*) denote the probability distribution of *i*.

$$ARI=\frac{2(ad-bc)}{\left(a+b)(b+d)+(a+c)(c+d\right)},$$ (3)

where *a* represents the number of point pairs belonging to the same cluster in both ground-truth and clustering results; *b* represents the number of point pairs belonging to the same cluster in ground-truth but not in clustering results; *c* represents the number of point pairs not belonging to the same cluster in ground-truth but in clustering results. *d* represents the number of point pairs that do not belong to the same cluster under both ground-truth and clustering results. The value range of ARI is [-1,1], and the value range of NMI is [0,1]. Larger values of ARI and NMI indicate better clustering results.

### 3.1 Benchmarking models

(1) **Nie’s work**: an improved non-federated k-means published in TKDE2022 [14].

(2) **k-FED**: SOTA method of federated clustering algorithm published in ICML2021 [21], and its parameters are *k* (k of k-means at the server) and kprime (the number of uploaded centers).

(3) **MUFC**: another SOTA method of federated clustering algorithm published in ICLR2023 [22], and its parameters are *k* (*k* of k-means at the server) and kprime (the number of uploaded centers).

### 3.2 Experimental results

**Fig 3:**
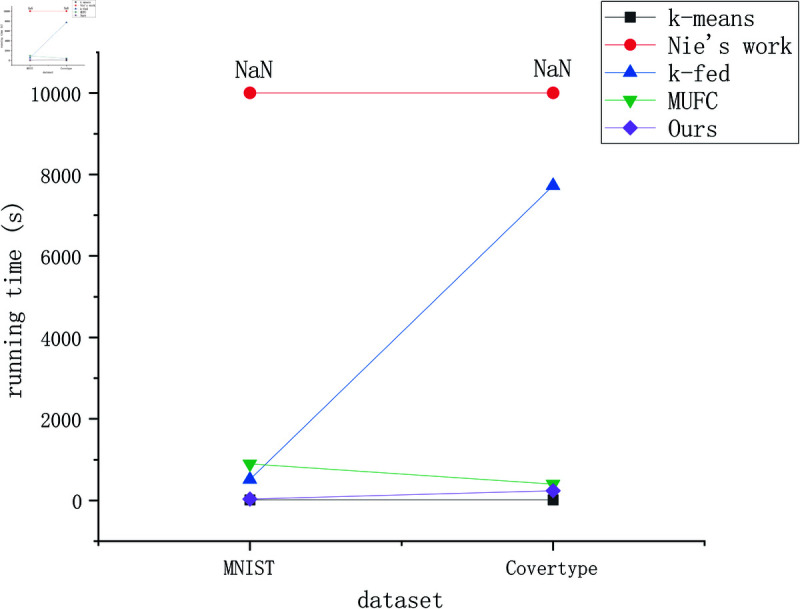
Running time comparison.

**Fig 4:**
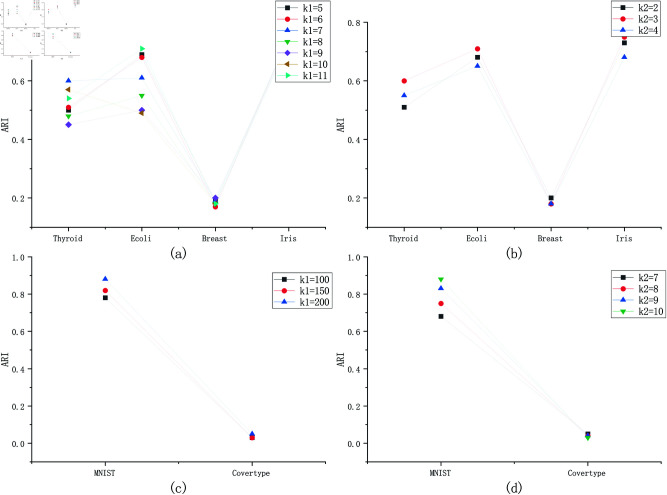
The influence of parameters*k1* and *k2* on clustering results.

**Fig 5:**
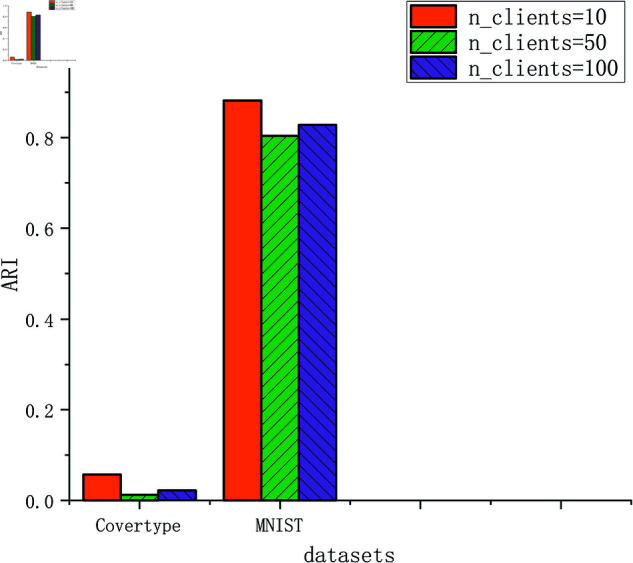
Clustering results under different numbers of clients.

The clustering results in terms of ARI and NMI are shown in Table 2 when the number of clients is 10. We compare the time of all algorithms using two large-scale datasets as shown in Fig 3. We show the influence of parameters *k1* and *k2* on clustering result in Fig 4, we also show the clustering results under different number of clients in Fig 5.

See Table 2, for k-FED, ARI=0.43(3/1) for dataset Thyroid, 3 denotes the parameter *k* (*k* of k-means at the server) and 1 denotes parameter kprime (the number of uploaded centers). For MUFC, ARI=0.58(3/3) for dataset Thyroid, 3 denotes the parameter *k* (*k* of k-means at the server) and 1 denotes parameter kprime (the number of uploaded centers). For **FKmeansCB**, ARI=0.60(7/3) for dataset Thyroid, 7 denotes the parameter *k1* (number of clusters in the clients), and 3 denotes parameter *k2* (number of clusters in k-means at the server). **FKmeansCB** performs better than k-FED and MUFC because **FKmeansCB** obtain better global centers.

See Table 3, we make ablation experiments to show the influence of parameters privacy budget (ϵ) on clustering results, where ϵ is an important parameter in Laplace noise. We can see that for most datasets, the impact of noise on clustering results is not particularly significant.

**Table 3:** The influence of parameters privacy budget (ϵ) on clustering results (ARI).

Datasets	ϵ=1	ϵ=5	ϵ=10	ϵ=20
Thyroid	0.64	0.62	0.62	0.60
Breast	0.22	0.20	0.19	0.20
Ecoli	0.45	0.69	0.74	0.71
Iris	0.88	0.82	0.79	0.75
Covertype	0.05	0.04	0.05	0.05
MNIST	0.88	0.88	0.88	0.88

## 4 Discussion

See Table 2, For the six selected datasets, FKmeansCB performs significantly better than other algorithms, especially the MNIST dataset, achieving very competitive clustering results. For large-scale datasets, Nie’s algorithm has exceeded the memory capacity, making it difficult for non federated clustering algorithms to handle large-scale datasets, while federated clustering algorithms have a natural advantage in handling large-scale data.

See Fig 3, In terms of clustering time, k-means has the least clustering time because its time complexity is the lowest. FKmeansCB has much less running time than k-FED and MUFC because the proposed algorithm has a time complexity of O(n+m+p), which has a significant advantage in time compared to similar federated clustering algorithms. Although the running time is longer than k-means (there is no order of magnitude difference between the two), the clustering accuracy is much better than k-means. Overall, the proposed algorithm has significant advantages in both running time and clustering accuracy.

See Fig 4, the influence of parameters *k1* and *k2* on clustering results is not significant, it is difficult to discover the patterns in it. For the k-means, the number of clusters has a significant impact on the clustering results. The selection of parameters for the k-means has always been very difficult. For the proposed algorithm FKmeansCB based on k-means, it is even more difficult to determine the parameter values (*k1* and *k2*). After more than 200 times of experiments, we have summarized the following rules, for small datasets, $\text{k1}\in [5,10],\text{k1}\in Z^{+}$, for big datasets, we can set *k2* to 150 or 200. The *k2* is easy to set, it equals to the number of ground-truth labels. See Fig 5, we also show the clustering results under different number of clients, the clustering results are close.

**Fig 6:**
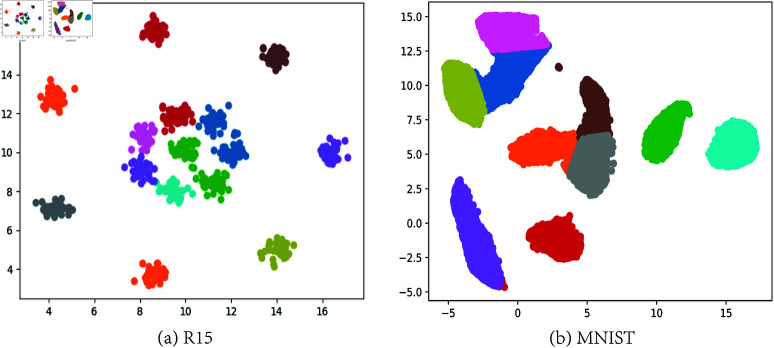
Clustering results on R15 and MNIST by using FKmeansCB.

See Fig 6, we presented the visual clustering results of the proposed algorithm FKmeansCB by using dataset R15 and MNIST.

## 5 Conclusions

In this paper, we propose a new federated k-means clustering algorithm. First, we run k-means clustering on the client side to obtain cluster centers, which faithfully represent the cluster backbone (*i.e.*, the data structures of the clusters). We then upload these cluster centers to the server. Subsequently, the server aggregates all cluster centers and runs the k-means clustering algorithm to obtain global cluster centers, which are then sent back to the client. Finally, the client assigns all data points to the nearest global cluster center to produce the final clustering results.

In the future, we will combine federated clustering and deep clustering and apply it to large-scale image clustering problems.
